# The Robustness of Pathway Analysis in Identifying Potential Drug Targets in Non-Small Cell Lung Carcinoma

**DOI:** 10.3390/microarrays3040212

**Published:** 2014-10-27

**Authors:** Andrew Dalby, Ian Bailey

**Affiliations:** Faculty of Science and Technology, University of Westminster, Westminster W1W 6UW, UK; E-Mail: I.Bailey@westminster.ac.uk

**Keywords:** NSCLC, pathway analysis, Reactome, robustness, squamous, adenocarcinoma

## Abstract

The identification of genes responsible for causing cancers from gene expression data has had varied success. Often the genes identified depend on the methods used for detecting expression patterns, or on the ways that the data had been normalized and filtered. The use of gene set enrichment analysis is one way to introduce biological information in order to improve the detection of differentially expressed genes and pathways. In this paper we show that the use of network models while still subject to the problems of normalization is a more robust method for detecting pathways that are differentially overrepresented in lung cancer data. Such differences may provide opportunities for novel therapeutics. In addition, we present evidence that non-small cell lung carcinoma is not a series of homogeneous diseases; rather that there is a heterogeny within the genotype which defies phenotype classification. This diversity helps to explain the lack of progress in developing therapies against non-small cell carcinoma and suggests that drug development may consider multiple pathways as treatment targets.

## 1. Introduction

In comparison to other cancers, over the last 30 years there has been little progress in the median survival time of lung cancer patients [1]. The median survival rate remains at around 3 months. Lung cancer cases account for about 13% of the total number of cancer cases diagnosed each year. It is also an increasingly significant cause of mortality in developing countries where there are a higher proportion of smokers [2]. Non-small cell lung carcinoma (NSCLC) is the most common form of bronchial tumor. Histological analysis of the tumors can divide them into two major sub-groups, adenocarcinoma and squamous cell carcinoma [3]. Some cases do not fall clearly into either of the sub-groups and so these are assigned to having a mixed morphology. Outside of these two main groupings there are also a small number of other rare histological groups.

Microarrays can contribute to the treatment and therapy of NSCLC in two important ways. The first is by allowing earlier detection of the tumors through the use of biomarkers [4]. The essential feature of a biomarker is that it must provide an accurate method for detecting the disease, by having a high sensitivity and high specificity. Another important factor is that biomarker detection should be non‑invasive. This is particularly important in lung cancer as you do not want to have to take samples of lung tissue itself and so biomarkers need to be available for either breath samples or blood samples. The second way that microarrays can contribute to the treatment of NSCLC is by identifying genes that are differentially expressed in the tumors and that could therefore be a target for new anti-cancer drugs. In this case the focus for the gene expression is the tumor tissue itself compared to normal lung tissue. Recently a number of large-scale studies have been under-taken to understand the factors involved in tumor progression [5,6,7]. These studies have used a variety of genomic, transcriptomic and proteomic methods and have identified, copy number variation, mutation and DNA methylation as well as changes in gene expression as differentiating between healthy lung tissue and tumors.

One of the past difficulties in identifying genes that are important in determining cancer progression (“oncogenes”) has been the large number of differentially expressed genes. Microarray data is often noisy, and there is almost always a lack of technical replicates [8,9,10] One of the best known examples of microarray analysis is the diffuse large B-cell lymphoma study by Alizadeh *et al.* [11]. The data from this study has been used in a number of subsequent re-analyses, which have produced differing results. One study even suggested that computationally the data was inadequate for resolving the problem of identifying the significant genes [12]. This is a fundamental problem when you have high dimensional data, where a large number of variables produce a small number of outcomes. In this case a large number of gene expression values contribute to a small number of phenotypes (either being a tumor cell or not being a tumor cell). As an absolute minimum the number of biological samples should to exceed the number of independent variables. In the case of microarrays the genes are not expressed independently and so removing genes that show high degrees of correlation, and genes that do not vary at all between all of the different phenotypes can reduce the number of variables. This is why it is usual to perform a gene filtering step in microarray analysis in order to reduce the number of genes that are considered for testing for differential expression [13,14]. The problem is that this filtering can be rather arbitrary and might have an effect on the results of the analysis [15,16]. It would be better to use the complete dataset with appropriate corrections for multiple statistical tests. Another factor in the processing of the microarray data that has been shown to affect the results is normalization of the samples. Once a set of differentially expressed genes has been identified these are often then reduced further by the use of gene set enrichment analysis (GSEA), to show which functional annotations are significantly up or down regulated [17]. An alternative to using GSEA, is to use a network based approach using data from biological pathways.

In this paper seven publicly available datasets for NSCLC are reanalyzed and a network based analysis is carried out using the pathways from the Reactome database [18].

## 2. Experimental Section

A search for NSCLC and organism Homo sapiens, in the ArrayExpress database yielded 223 datasets [19]. The search was then refined to only include Affymetrix data. This reduced the number of datasets to 115. From these six datasets were chosen where the study looked only at NSCLC, it was a transcriptome profiling array and the number of samples was above 40. The datasets used in the study are listed in Table 1. In total there are 669 arrays in the combined data.

**Table 1:** Datasets from ArrayExpress used in the data analysis.

Accession	Title	Number of Arrays	Date
E-GEOD-6044 [20]	Transcription profiling of human lung cancers	47	14 June 2008
E-GEOD-18842 [21]	Transcription profiling of NSCLC	91	1 October 2010
E-GEOD-19188 [21]	Transcription profiling of human lung cancer	156	28 May 2010
E-GEOD-40275 [22]	Gene expression of normal lung tissue and patients with SCLC or NSCLC	84	25 August 2012
E-GEOD-43458 [23]	Gene expression profiling of lung adenocarcinomas and normal lung	110	6 August 2013
E-GEOD-50081 [24]	Validation of histology independent prognostic signature for early stage NSCLC	181	22 September 2013

All of the analysis was carried out using R version 3.1.0 and Bioconductor version 2.14, using R‑studio version 0.98 as a graphical user interface [25,26]. Quality checking of the arrays was not carried out, as the objective of the study was to test the robustness of the analysis. The data from HGU133aplus2 and HGFocus arrays was normalized using rma, gcrma and farms as described previously [16]. The E-GEOD-40725 is an exon dataset and could only be normalized using rma and the E-GEOD-43458 dataset could only be normalized with rma and farms. This is because feature tables for the other normalization methods are not available for some array designs. After normalization differential expression was calculated using limma for the multiple testing correction and Bayesian fitting on the complete dataset [27]. Pathway analysis was carried out against the Reactome database using ReactomePA [28].

## 3. Results and Discussion

**Figure 1:**
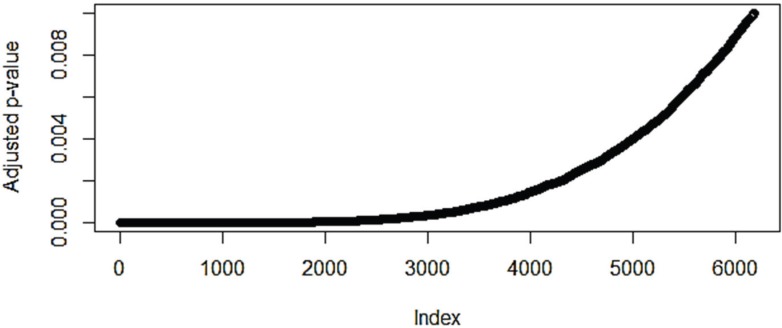
A plot of the adjusted *p*-values for the top scoring probes from the differential expression analysis between adenocarcinoma and squamous cell carcinoma in the E-GEOD-50081 dataset.

Some of the datasets had a very large number of differentially expressed genes. In the cases of E-GEOD-19188, E-GEOD-40725 and E-GEOD-50081 it was necessary to reduce the number of differentially expressed genes before running ReactomePA as the large number of genes had a negative effect on the algorithm finding any enriched pathways. In the cases of the E-GEOD-18842 and EG-GEOD-43458 datasets although there are a large number of differentially expressed genes these come from a small number of pathways, and so the pathway analysis could be carried out without the use of a cut-off. A plot of the adjusted *p*-value for the differential expression shows a long almost horizontal region before a sharp rise and then a steady linear increase (Figure 1).

From the graph the point where the adjusted *p*-value starts to rise from the horizontal is around 2000 probes and so this was used as a limit on the number of differentially expressed genes in the problem datasets, except in the case of the normal *vs.* small-cell carcinoma comparison in dataset E‑GEOD-40725 where a cut-off of 1500 was needed.

### 3.1. The Effect of Normalization on the Number of Differentially Expressed Genes

Table 2 shows the numbers of differentially expressed probes and genes for the different datasets. In some datasets there are multiple sub-groups and so these datasets have multiple comparisons in order to determine the variation in expression levels. It is also important to note that the number of genes identified by their EntrezID number is larger than the number of probes identified on the array. This is because there is a one-to-many relationship between the probes and EntrezID. This is far from ideal as this means that there are cross-gene effects in the array.

From the table it is clear that the method used for normalization makes a difference to the number of differentially expressed genes and sometimes this can be quite large, amounting to over 1000 genes or about 20% of the total number. This could have a significant impact on any subsequent gene set enrichment analysis.

There is also a general trend to a larger number of differentially expressed genes in the larger datasets. This reflects the higher power (improved sensitivity) of larger datasets, and the much smaller E‑GEOD-6044 has a particularly small number of variable genes. However, the *p*-value distribution (Figure 1) also suggests that in the larger datasets noise is becoming an issue and that there are a large number of supposedly differentially expressed genes might be an artifact of those datasets. The E‑GEOD-18842 and E-GEOD-43458 datasets both perform very well in the subsequent pathway analysis compared to the datasets where it was necessary to introduce a cut-off to reduce the number of genes being considered. Both of those datasets contained only two sub-groups and the division of the data into further sub-groups unless these were part of the original experimental design is highly controversial and likely to result in an increased number of false positives. Only the E-GEOD-50081 dataset suggests that there are a large number of differentially expressed genes between adenocarcinoma and squamous cell carcinoma, but this dataset was specifically created in order to ask this question.

**Table 2:** The number of differentially expressed probes and genes (EntrezIDs) between the two specified conditions for each of the datasets normalized using rma, gcrma and farms. In cases where the cut-off of 2000 probes was used the number of EntrezIDs are given for these cut-off values.

Dataset	Conditions	Number of Probes			Number of EntrezIDs		
		rma	gcrma	farms	rma	gcrma	farms
E-GEOD-6044	Normal-Adenocarcinma	235	260	196	251	293	209
	Normal-Small	556	554	482	591	603	516
	Normal-Squamous	341	347	248	368	388	264
	Adenocarcinoma-Squamous	763	556	579	821	593	622
E-GEOD-18842	Normal-NSCLC	6497	5951	5520	6481	6028	5599
E-GEOD-19188	Healthy-Tumor	29,727	20,904	17,242	31,998	22,636	18,741
	Healthy-Tumor	2000	2000	2000	2135	2132	2110
E-GEOD-40275	Normal-Adenocarcinoma	13,255			16,387		
		2000			2418		
	Normal-Small Cell	14,942			18,559		
		1500			1947		
	Normal-Metastatic	7132			8897		
		2000			2492		
	Normal-Squamous	11,543			14,339		
		2000			2455		
	Adenocarcinoma-Squamous	274			362		
	Adenocarcinoma-Metastatic	6619			8278		
		2000			2455		
E-GEOD-43458	Normal-Adenocarcinoma	12,800		7099	14,186		7734
E-GEOD-50081	Adenocarcinoma-Squamous	7769	6227	6181	8393	6728	6643
		2000	2000	2000	2121	2132	2148
	Adenocarcinoma-Mixed	231	437	168	249	463	186
	Squamous-Mixed	1	42	0	1	44	0

### 3.2. Pathway Analysis

**Figure 2:**
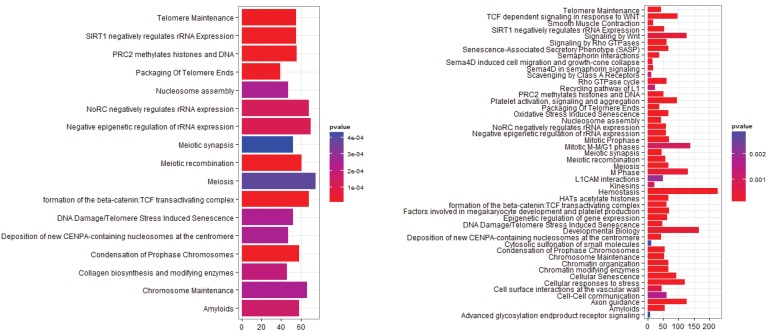
Pathway Analysis for E-GEOD-43458, Normal-Adenocarcinoma. (**left**) After rma normalization; (**right**) After farms normalization.

A pathway enrichment analysis of the differentially expressed genes was carried out against the Reactome pathway database using the ReactomePA module. Like gene set enrichment analysis this produces a list of key terms, in this case pathways, and a *p*-value for the probability that the observed distribution of expression occurred by chance. The cut-off value for the pathway p-values was chosen at 0.05. An example of the effect of normalization on the pathway analysis is given in Figure 2.

In this case there are a large number of additional pathways after farms normalization, but the pathways identified by the rma normalized analysis are conserved in the farms normalized results. It is possible that in this case farms normalization produces a dataset that is more sensitive to changes in pathway regulation, but it is also possible that a number of these pathways will be false positives.

#### 3.2.1. Pathways that Are Differentially Expressed between Normal Lung Tissue and Tumors

Two of the datasets E-GEOD-18842 and E-GEOD-19188 compare normal lung tissue to NSCLC or tumor tissue, respectively. Of these E-GEOD-18842 has a much better defined list of differentially expressed genes. Again this is going to partly be a result of the larger size of the dataset as this results in a much lower standard error in the expression levels of the comparison groups and an increase in power of the experiment, but noise is also a factor as the final list contains almost all of the genes on the array. A cut-off had to be used for E-GEOD-19188 and only the top 2000 genes were used for pathway analysis.

**Figure 3:**
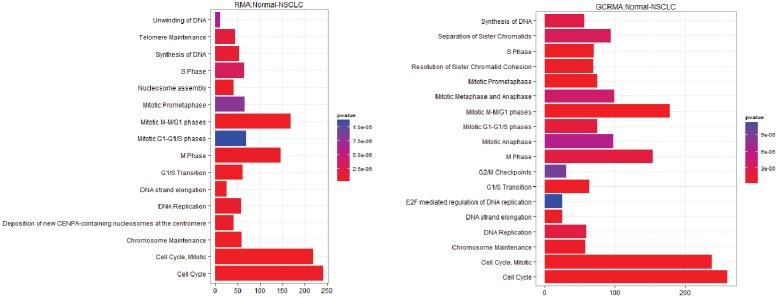
Pathway Analysis for E-GEOD-18842, Normal-NSCLC (**left**) After rma normalization; (**right**) After gcrma normalisation.

From the E-GEOD-18842 the three different normalization methods produce a very similar pathway analysis. The rma and farms normalized data give identical pathway analysis and identify 16 significant pathways (Figure 3). The results for the gcrma normalized data contain 12 out of the 16 previously identified pathways: deposition of new CENPA-containing nucleosomes at the centromere; telomere maintenance; unwinding of DNA and nucleosome assembly are absent, and separation of sister chromatids; resolution of sister chromatid cohesion; E2F mediated regulation of DNA replication; mitotic metaphase and anaphase; mitotic anaphase; DNA strand elongation and G2/M checkpoints are added.

**Figure 4:**
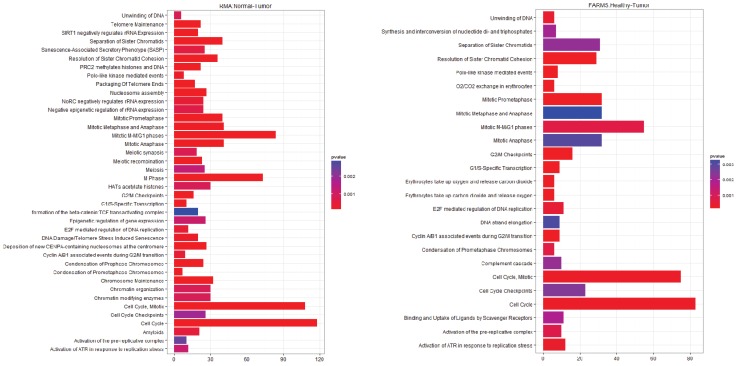
Pathway Analysis for E-GEOD-19188, Healthy-Tumor (**left**) After rma normalization; (**right**) After farms normalization.

The results from E-GEOD-19188 are less definitive (Figure 4). From the rma and gcrma normalized data over 40 pathways are identified as significant. There is considerable overlap between these two pathway sets with 37 of the top 40 pathways being conserved between the two analyses. The farms normalized data only yields 29 significant pathways and some of those identified are notably different from the other results, especially the pathways associated with erythrocytes that are absent from the other pathway analyses (O_2_/CO_2_ exchange in erythrocytes, hemostasis, erythrocytes take up oxygen and release carbon dioxide, erythrocytes take up carbon dioxide and release oxygen). Associations between hemostasis, platelets and cancer were proposed in the 1980s, but these associations have only recently become the focus of renewed attention for pathway analysis [29,30,31].

The overlapping pathways between the E-GEOD-18842 and E-GEOD-19188 contain many of the usual suspects, such as the control of mitosis and control of the cell cycle including checkpoints. The E2F mediated regulation of DNA replication has also been identified as important in a number of different cancers, as this transcription factor regulates cyclin E [32]. Two other pathways of interest are the Polo-like kinase mediated events and the activation of ATR in response to replication stress. Polo‑like kinase (PLK1) is a DNA damage checkpoint and it has been a target for cancer therapy [33,34]. Ataxia-telangiectasia and Rad3-related protein (ATR) halts DNA replication when replication forks are stalled and need to be repaired. The absence of gene creates fragile sites in the chromosome. ATR has been targeted as a possible cancer preventative or in boosting the effectiveness of existing therapies [35,36].

#### 3.2.2. Pathways that Are Differentially Expressed between Normal Lung Tissue and Adenocarcinoma

Three datasets have a comparison between normal lung tissue and adenocarcinoma: E-GEOD-6044, E-GEOD-40725 and E-GEOD-43458. Of these the E-GEOD-43458 dataset is the largest and it is the only one that was specifically targeted to adenocarcinoma.

The smallest of these datasets is E-GEOD-6044. This array also used the hgfocus gene array and not the more complete hgu133a array. From the rma and farms normalized data less than 10 pathways were identified and these were: biological oxidation; axon guidance; hemostasis; platelet activation, signaling and aggregation; platelet degranulation; translation of GLUT4 to the plasma membrane; response to elevated platelet cytosolic Ca2^+^; L1CAM interactions; phase 1-functionalisation of compounds; membrane trafficking and extracellular matrix organization. It is noteworthy that none of these pathways were amongst those identified as differences between the normal and tumor tissues and that they are not involved in cell cycle or DNA regulation. Phase 1-functionalisation is the processing and export of proteins from the endoplasmic reticulum and the reactions involve cytochrome P-450. Sugar transporters have previously been identified as having a role in cancer [37]. The L1 cell adhesion molecule L1CAM is associated with invasive tumors and metastasis [38,39]. After gcrma normalization pathway analysis identifies over 40 significant pathways (Supplementary Figure S1). These include the DNA regulation, mitotic pathways and cell cycle pathways previously identified in the normal-tumor comparisons. It also includes the meiosis pathways, negative regulation of rRNA expression and amyloids.

**Figure 5:**
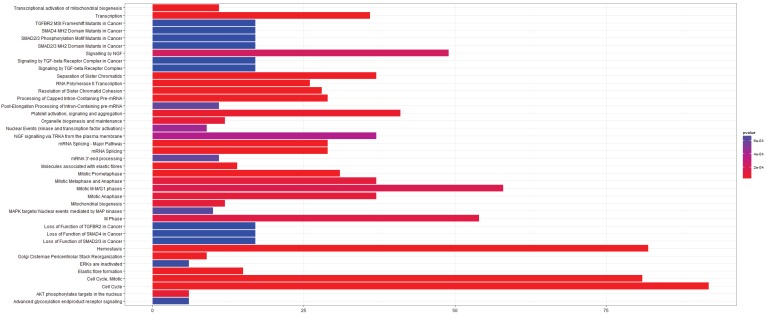
Pathway Analysis for E-GEOD-40275, Normal-Adenocarcinoma.

The E-GEOD-40275 dataset uses an exon array and so it can only be normalized using rma. The results of pathway analysis are very different from those for the E-GEOD-6044 dataset and the normal‑tumor analysis. These include a number of cancer specific pathways involving mutants and also a group of pathways involved in mRNA regulation and processing (Figure 5).

The E-GEOD-43458 dataset could only be normalized with rma and farms as gcrma is incompatible with the array type used. The results from the pathway analysis have been shown in Figure 2. As stated above there is a large difference in the number of pathways identified between the two analyses, with many more differentially expressed pathways after farms normalization. Many of them are previously identified pathways involved in cell cycle regulation and mitosis. Amyloids appear in both of the pathway analyses and they were identified in the gcrma normalized pathway analysis of E-GEOD-6044. Serum amyloid a protein has been known for some time to be a biomarker for lung cancer and nasopharyngeal cancer [40,41]. Signaling pathways including the Rho GTPases, Wnt and TCF are also present in the post-farms pathway analysis. These signaling pathways have previously been associated with cancer, although TCF has mainly been associated with colorectal cancers [42,43,44,45,46]. The L1CAM pathway is also identified as being significant in agreement with the results from E-GEOD-6044.

#### 3.2.3. Pathways that Are Differentially Expressed between Normal Lung Tissue and Squamous Cell Carcinoma

Two datasets are available to compare the transcriptomes of normal lung tissue and squamous cell carcinoma, E-GEOD-6044 and E-GEOD-40275. The E-GEOD-6044 data shows a similar profile to the normal-adenocarcinoma pathway profile for E-GEOD-40275 including the TCF dependent signaling in response to Wnt (Supplementary Figures S2–S4). There are also shared pathways with the normal-adenocarcinoma analysis of the same dataset. The results suggest that squamous cell carcinoma has an affect on a more diverse range of pathways than adenocarcinoma.

**Figure 6:**
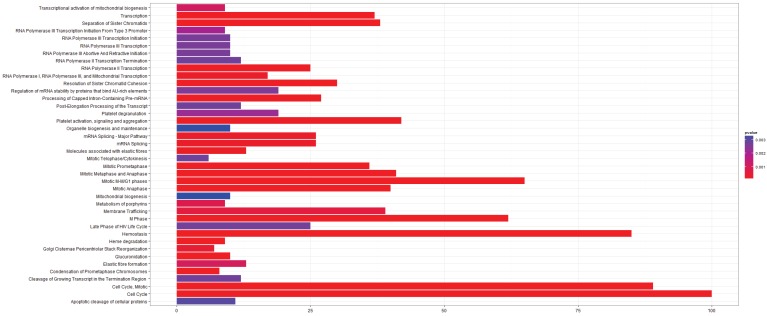
Pathway analysis for E-GEOD-40275, Normal-Squamous Cell Carcinoma.

The E-GEOD-40275 normal-squamous cell carcinoma comparison identifies platelet associated pathways as well as RNA processing as the most important groups of differentiated pathways (Figure 6). These overlap with the pathways identified in the normal-adenocarcinoma comparison carried out with the same dataset and also the normal-adenocarcinoma pathway differences identified using the E-GEOD-6044 data.

#### 3.2.4. Pathways that Are Differentially Expressed between Adenocarcinoma and Squamous Cell Carcinoma

From the previous analysis it is apparent that there is considerable overlap in the pathways that are differentially expressed in adenocarcinoma and squamous cell carcinoma. There are three datasets that allow direct comparison between the transcriptomes of the two NSCLC sub-types, E-GEOD-6044, E-GEOD-40275 and E-GEOD-50081 (this dataset was specifically created to compare gene expression between cancer sub-types). Table 2 shows that there are only a small number of differentially expressed genes between the two NSCLC sub-types except in the E-GEOD-50081 dataset. It is therefore slightly surprising that after pathway analysis this large number of genes reduces to 13 pathways: unwinding DNA; type I hemidesmosome assembly; telomere maintenance; SIRTS1 negatively regulates rRNA expression; packaging of telomere ends; nucleosome assembly; metabolism of porphyrins; heme degradation; glucuronidation; DNA strand elongation; Deposition of new CENPA-containing nucleosomes at the centromere; condensation of prophase chromosomes and chromosome maintenance.

The E-GEOD-6044 dataset has many fewer differentially expressed genes (although more than are differentially expressed between normal-adenocarcinoma and normal-squamous cell carcinoma). This produces a much more extensive list of pathways, with novel pathways involved in extra-cellular processes, such as extracellular matrix organization, cell-cell communication, cell-cell junction organization and immune system. The data however is very noisy and there is poor overlap between the results from pathway analysis after the three different normalization methods and so these results should be considered with some caution.

Finally the E-GEOD-40275 also shows a small number of differentially expressed pathways. These agree with the extra-cellular pathways found in the E-GEOD-6044 results lending support to those findings, and also include new pathways involved in fatty acid triacylglycerol, and ketone body metabolism, as well as the metabolism of lipids and lipoproteins. There are also two pathways highlighted that are involved in the regulation of the peroxisome proliferator-activated receptor alpha (PPARA). There is some evidence that PPARA is associated with breast cancer but this is the first time it has been identified as involved in lung cancer although the gamma receptor has previously been identified and playing a role in inhibiting lung cancer cell growth [47,48].

## 4. Conclusions

The analysis shows that there is a clear genetic demarcation between healthy lung tissue and NSCLC which survives different datasets and methods of normalization. The results for the different NSCLC phenotypes (adenocarcinoma and squamous cell carcinoma) are much less clear. A comparison of the pathways which are overrepresented between the NSCLC phenotypes with normal lung tissue provides a clear core of altered cellular functions. However, when comparing the NSCLC phenotypes themselves, it is not possible to draw a clear and consistent delineation between their transcriptomes across all of the datasets. Most of the common pathway differences between the NSCLC phenotypes concern extra-cellular processes, and these are more likely to be associated with metastasis than phenotype differentiation. A possible distinguishing feature is the lipid biosynthetic pathways identified in the E-GEOD-40275 dataset, but these pathways were not reproduced in the other datasets. The small numbers of pathways that seem to distinguish adenocarcinoma and squamous cell carcinoma suggests that the histological differences might not be as strongly represented at the genetic level. This result agrees with a previous study that has shown that there are distinct sub-groups within adenocarcinoma and a second study that suggested that there might be overlap in gene expression between the adenocarcinoma and squamous cell carcinoma groups, such that alternative sub-classes to those identified by histology might exist at the genetic level [16,49].

Many of the pathways that have been identified as targets in this study have previously been associated with cancer, either in lung cancer or in other tissues. These include the regulation of cell cycle and mitosis. Regulation of rRNA expression by SIRT1 and of histone methylation by PRC2 as well as the WNT signaling pathways are specific targets that distinguish healthy and tumor cells. The Amyloid pathway is an interesting additional target, because of its association with other age related diseases.

Future experiments need to focus on including a larger number of participants in order to sample disease diversity more effectively. Current studies have also not considered experimental repeats to reduce the effects of between sample variations. These are important when between subject variability is high because a component of this might be due to problems with experimental precision. In addition to new experimental results a complete meta-analysis of all of the available NSCLC data would improve the statistical power and reliability in predicting differentially expressed genes and pathways. This analysis would include the large datasets that are available from the Cancer Genome Atlas, which contain data for another 1000 cases. However, a serious challenge in incorporating these data is that they come from a number of alternative platforms and so combining them is difficult.

## Supplementary Files

Supplementary File 1
