# Supplementary material for: The Robustness of Pathway Analysis in Identifying Potential Drug Targets in Non-Small Cell Lung Carcinoma
Source: Microarrays (Basel). 2014 Oct 27;3(4):212–25. doi: 10.3390/microarrays3040212 (PMC4979055; doi:10.3390/microarrays3040212)

Supplementary Materials

The Robustness of Pathway Analysis in Identifying Potential Drug Targets in Non-Small Cell Lung Carcinoma

Andrew Dalby and Ian Bailey

Figure S1. The pathway analysis between normal and adeoncarcinoma in the gcrma normalized G-GEOD-6044 dataset.

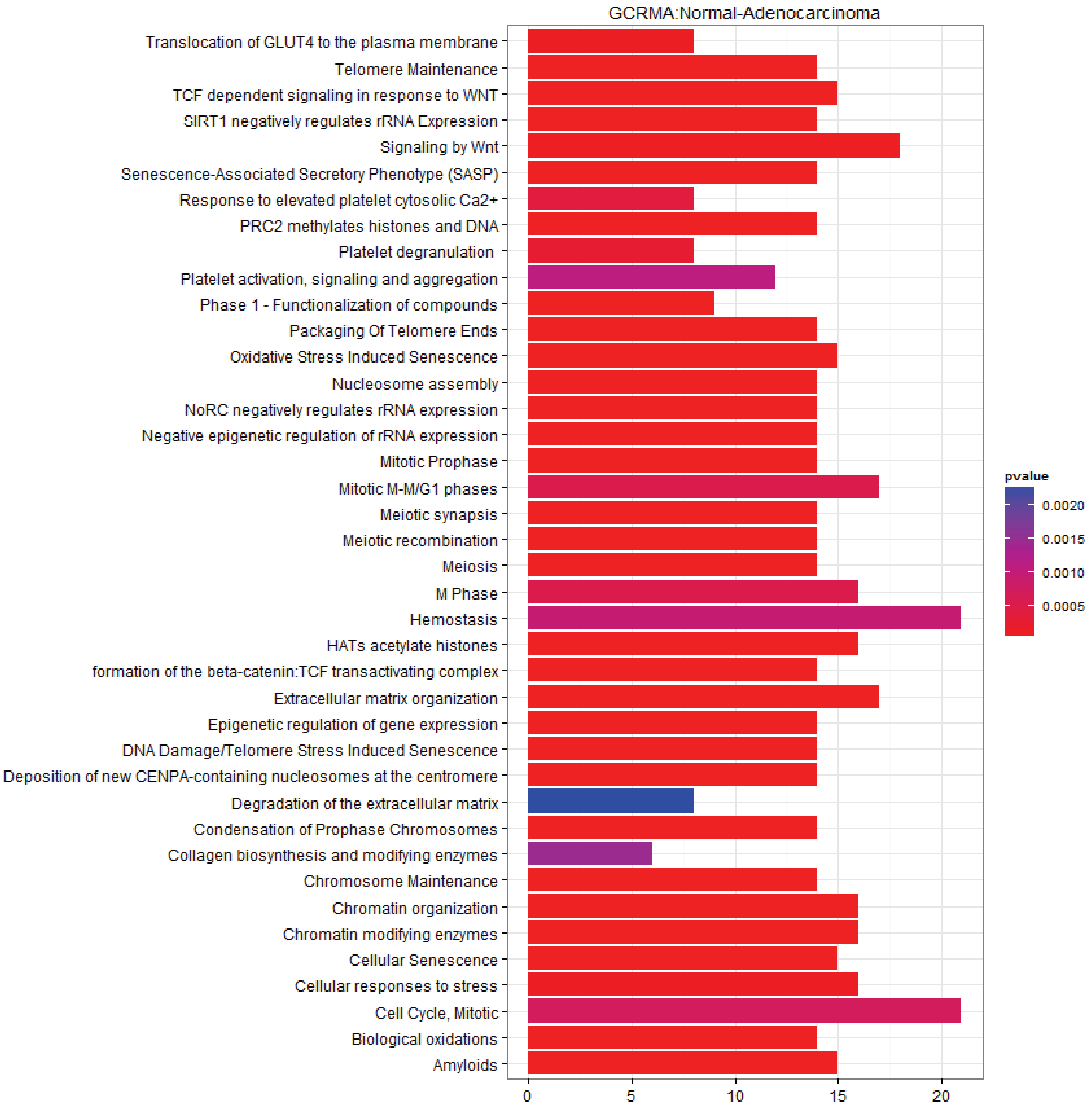

Figure S2. The pathway analysis between normal and squamous cell carcinoma in the rma normalized G-GEOD-6044 dataset.

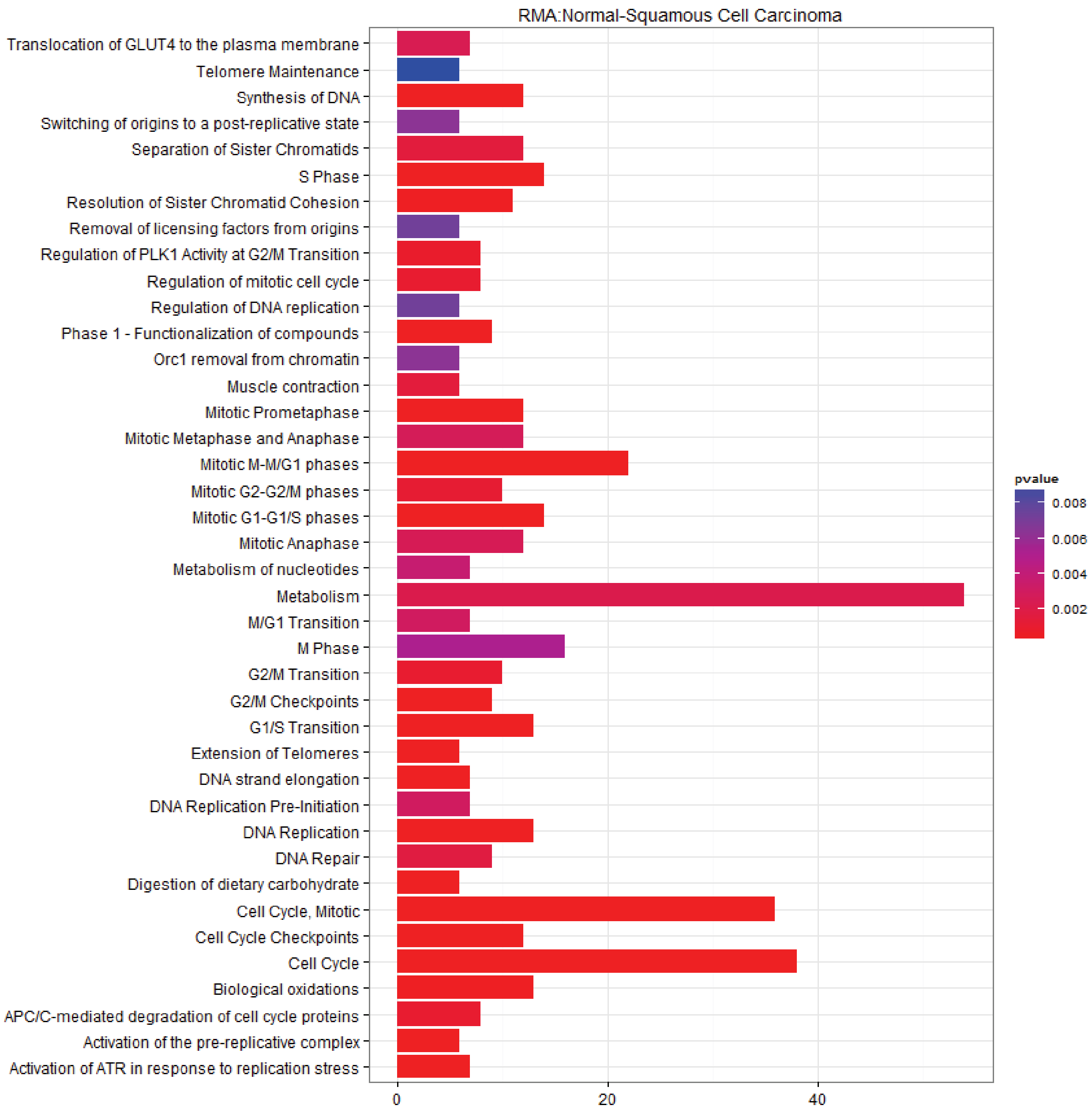

Figure S3. The pathway analysis between normal and squamous cell carcinoma in the gcrma normalized G-GEOD-6044 dataset.

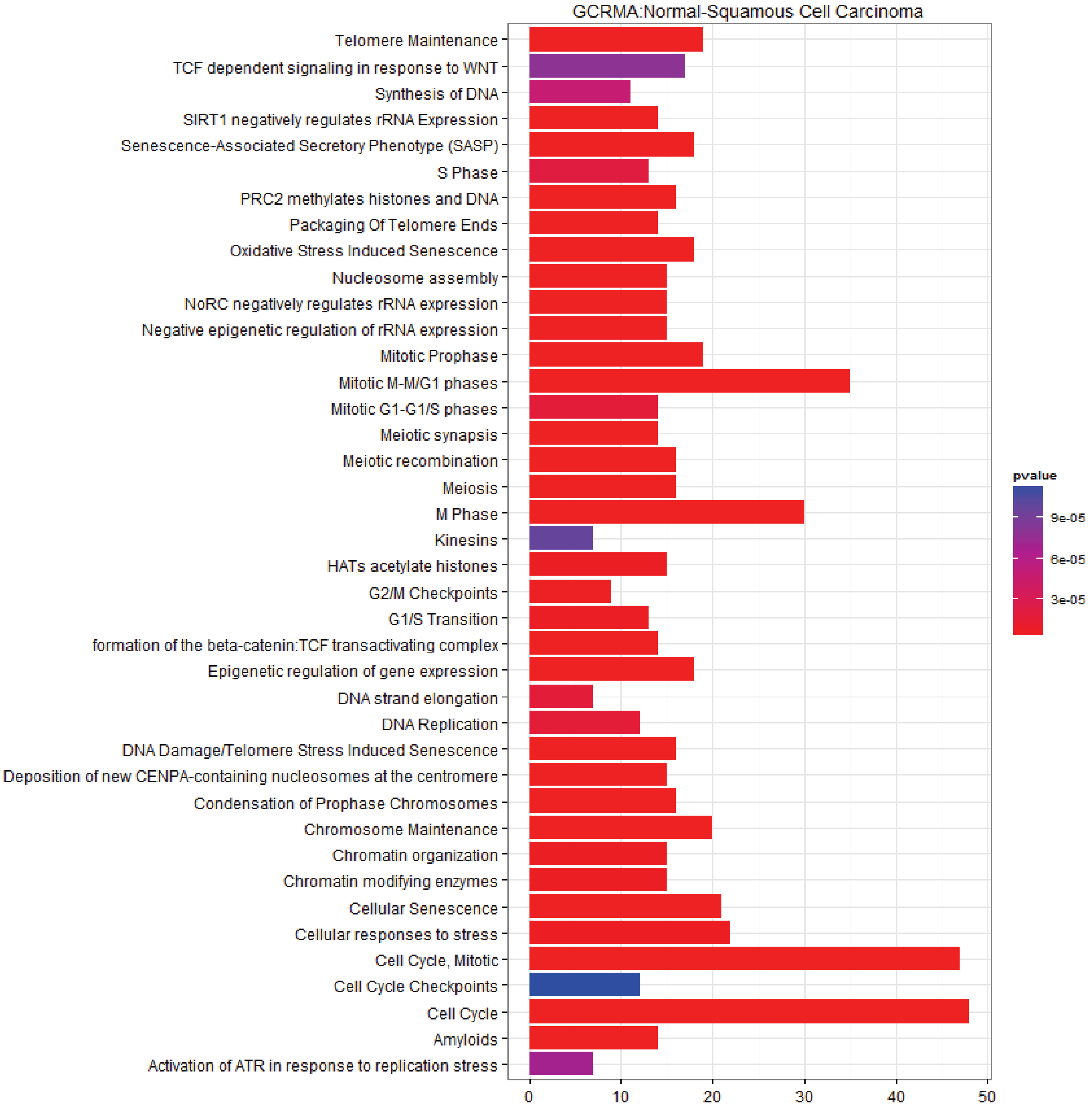

Figure S4. The pathway analysis between normal and squamous cell carcinoma in the farms normalized G-GEOD-6044 dataset.

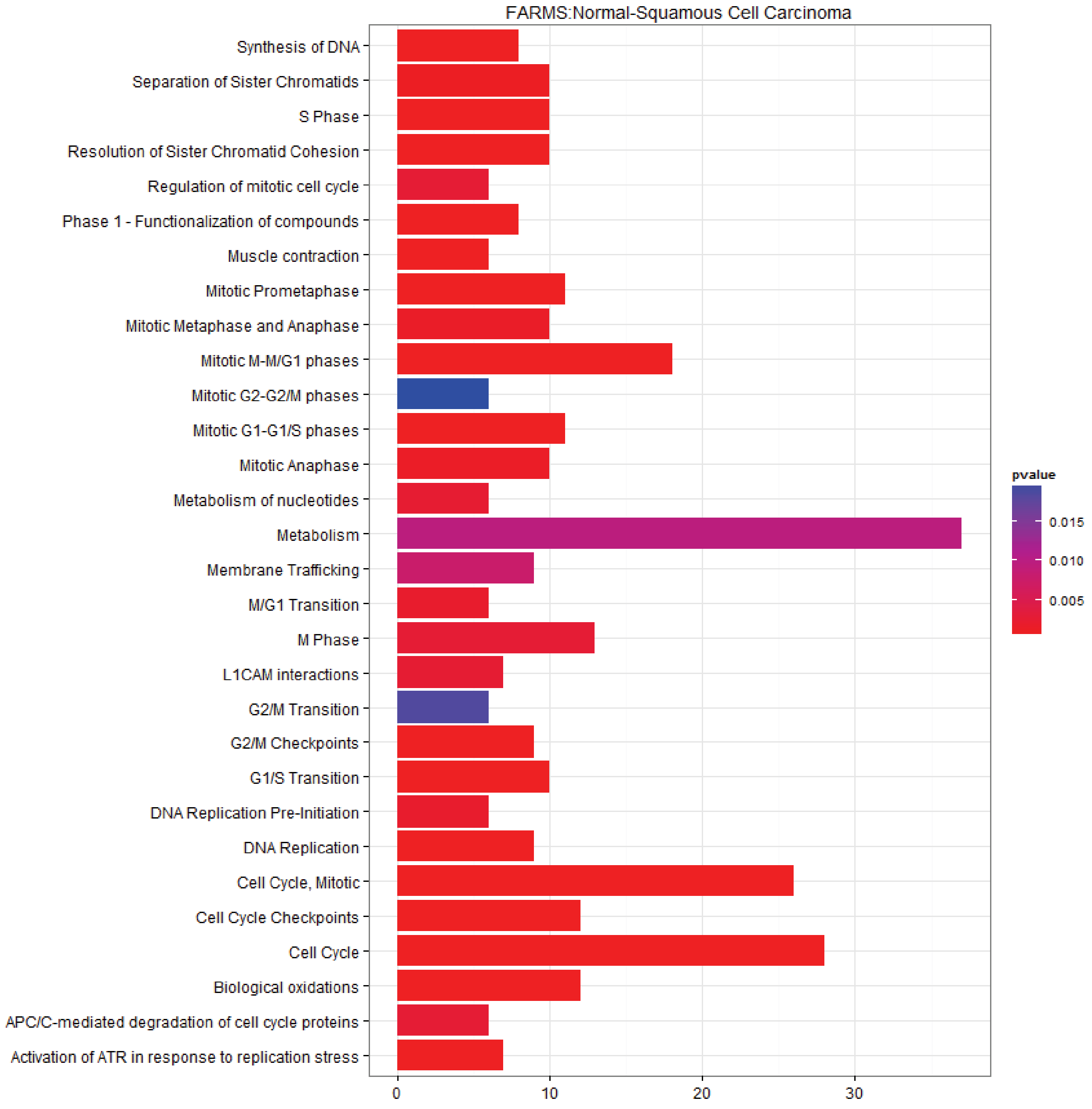

Supplement: Supplementary File 1 [file microarrays-03-00212-s001.pdf]
